# Interactions between extracellular vesicles and hormones: review

**DOI:** 10.5713/ab.25.0249

**Published:** 2025-08-25

**Authors:** Yiwen Bi, Yang Yang, Linsheng Gan, Wenhui Huang, Xin Feng, Huihua Zhang, Jie Liu, Limin Wei, Qien Qi

**Affiliations:** 1 School of Animal Science and Technology, Foshan University, Foshan, China; 2 Institute of Animal Science and Veterinary Medicine of Hainan Academy of Agricultural Sciences, Haikou, China; 3 Sanya Institute, Hainan Academy of Agricultural Sciences (Hainan Experimental Animal Research Center), Sanya, China

**Keywords:** Extracellular Vesicles, Hormones, Interaction

## Abstract

Extracellular vesicles (EVs) are important in both healthy and unhealthy states, and this review looks at how they interact with hormones. As key messengers of intercellular communication, EVs are rich in a variety of bioactive molecules and play a significant role in the *in vivo* environment. Hormones, as core mediators of organismal functions, affect the production and release of EVs through various signaling pathways, thereby regulating the function of target cells. Conversely, EVs can also carry biologically active molecules that significantly influence hormone synthesis and secretion. In this review, we discuss the effects of different hormones (such as estrogen, testosterone, and follicle-stimulating hormone) on EVs, as well as the regulatory effects of EVs on hormone secretion. This reveals the potential clinical applications of this field, including the possibility as a biomarker. To further the creation of novel diagnostic instruments and treatment approaches, additional research into the molecular mechanisms underpinning this relationship is require.

## INTRODUCTION

Extracellular vesicles (EVs), as key messengers of intercellular communication, include various subtypes such as exosomes, microvesicles, and apoptotic bodies. They are rich in bioactive molecules such as proteins, nucleic acids, and lipids, which can play important roles in the *in vivo* environment [1]. At the same time, hormones, as core mediators in the precise regulation of biological functions, have long been considered the bridge connecting the functions of various systems. In recent years, the interaction between EVs and hormone regulatory network has gradually emerged, demonstrating the deep integration of EVs and hormone regulatory network, which is crucial for deepening the understanding of physiological and pathological mechanisms.

Hormones can affect the production and release of EVs in a variety of ways and further regulate the functional responses of target cells. For example, signal transduction pathways triggered by specific hormones can stimulate the synthesis and secretion of EVs, thereby transmitting molecular signals locally or remotely [2]. In addition, hormones can alter the cargo within EVs, such as the composition of miRNA, mRNA, or other proteins, affecting the post-transcriptional regulatory mechanisms of the recipient cells [3].

This dynamic interaction not only but also may provide insights for the development of novel therapeutic strategies.

Under pathological conditions, such as inflammation and tumor development, hormone-mediated changes in EVs are often accompanied by the appearance of specific markers, indicating the trajectory of the disease progression [4]. These characteristic EVs and their contents are considered as potential non-invasive biomarkers for early diagnosis, treatment monitoring, and even prognosis. At the same time, EV-based drug delivery systems are gradually demonstrating therapeutic potential. The use of hormone-modulated vesicles as carriers can accurately target the disease sites, improve drug efficacy, and reduce side effects, opening up a new direction in personalized medicine.

The existing reviews mainly focus on the regulation of EVs by hormones or the indirect effects of EVs on hormones [5,6]. This review further integrates the feedback regulation mechanisms of EVs on hormone synthesis and secretion, and systematically elaborates on the bidirectional interaction between them in reproduction, metabolism, and diseases, thereby providing a more comprehensive perspective for research in this field. The regulatory mechanisms of reproductive endocrinology (estrogen, anti-Müllerian hormone [AMH], etc.), stress response (cortisol, GH, etc.), and certain metabolic hormones (insulin) are highlighted. It should be noted that EVs regulation of neuroendocrine hormones (melatonin [MT], thyroid hormone) and the complete metabolic hormone spectrum (leptin, adrenocorticotropic hormone [ACTH], etc.) is still only sparsely studied. The following discussion will show the mutual influence of different hormones and EVs, based on the existing literature. In this paper, EVs are abbreviated as EVs to optimize the presentation.

## OVERVIEW OF EXTRACELLULAR VESICLES

EVs originate from the life activities of cells. Their formation is involved in a variety of cellular events, and they are divided into several categories according to different biogenesis pathways and properties. These mainly include exosomes, microvesicles, and apoptotic bodies. The main types of EVs are briefly described below. The content of this paragraph will be summarized in Table 1.

### Exosomes

Exosomes are derived from multivesicular bodies (MVBs) formed by endocytosis. When MVBs fuse with the cell membrane, their internal vesicles are released into the extracellular environment to form mature exosomes [7]. This process is subject to a series of meticulous regulations involving endosomal sorting mechanisms. Their diameter is about 40–150 nm [8], and they are rich in unique lipids, proteins, and nucleic acid molecules, such as tetraspanins, heat shock protein 70 (HSP70), and various microRNAs [9]. In addition, exosomes participate in intercellular signal transmission, affect the physiological state of adjacent or distal cells, and promote immune responses, nerve signal transduction, tumor microenvironment remodeling, and other processes [10].

### Microvesicles

Microvesicles are mostly 100–1,000 nm in diameter and are formed by budding of the cell membrane; their membrane composition is similar to that of the mother cell. The formation of microvesicles is associated with cell activation, injury, or stress [11]. At the same time, the formation of microvesicles is related to the asymmetric distribution of phospholipids in the cell membrane: the outer leaflet is rich in phosphatidylcholine and sphingomyelin, while the inner leaflet is mainly composed of phosphatidylserine and phosphatidylethanolamine. This asymmetry is maintained by flippase, floppase, and phospholipid scramblase. Calcium influx activates phospholipid scramblase, disrupts phospholipid asymmetry, and promotes membrane budding. Ca^2+^-dependent proteolytic degradation of the membrane–cytoskeleton linkage assists in budding. In addition, *ARRDC1* recruits ESCRT proteins *TSG101* and VPS4 to the cell membrane to promote budding. Vesicle budding has also been considered one of the mechanisms for the repair of plasma membrane damage [12].

### Apoptotic bodies

In the process of apoptosis, the cytoplasm is divided and packaged into large vesicles, which are eventually released from the main body of the cell into the extracellular space, thus forming apoptotic bodies [13]. When cell apoptosis occurs, the cell membrane shrinks and invaginates, wrapping cytoplasm, organelles, and nuclear debris, and forming vesicular bodies with a diameter of 500–2,000 nm (50–5,000 nm in some literature) [14,15]. Apoptosome plays a key role in the process of clearing senescent or damaged cells, avoiding inflammatory response and maintaining tissue stability.

## OVERVIEW OF HORMONES

Hormones, as a class of important signaling molecules in organisms, bear the significant responsibility of remotely regulating of the body’s physiological functions. They maintain the harmonious operation of the internal and external environments of the body through precise signal transduction mechanisms. According to their different chemical properties and mechanisms of action, hormones can be roughly classified into the following categories.

### Peptide hormones

These hormones are mainly composed of amino acid chains, including the well-known insulin, ACTH, and various growth hormones released by the pituitary gland. They exert diverse physiological effects in the body, such as regulating blood glucose levels, controlling energy homeostasis and metabolism, and participating in stress responses [16,17]. Peptide hormones bind to specific receptors on the membranes of target cells and trigger the conformational change in the receptors, then activate G proteins, enzyme-linked receptors, and other signaling molecules, initiating intracellular second messenger systems (such as cAMP, Ca^2+^, etc.), ultimately regulating gene expression or cell function to achieve the transmission and amplification of physiological effects [18].

### Steroid hormones

Steroid hormones are hydrophobic molecules, typically represented by testosterone, estrogen, progesterone, and glucocorticoids (e.g., hydrocortisone). Steroid hormones can freely penetrate the cell membrane due to their lipid-soluble properties. They first bind to specific receptors in the cytoplasm to form hormone-receptor complexes, which then enter the nucleus to affect gene transcription and regulate protein synthesis [19]. This process exert long-lasting effects on reproductive, metabolic, immune, and other functions. It is important to note that steroid hormone receptors can be either present in the cytoplasm or immobilized in the nucleus, depending on the specific hormone and cell type [20].

### Amino acid derivative hormones

These include thyroxine (T3, T4), catecholamines (such as epinephrine and norepinephrine) released from the adrenal medulla, and so on. The former is based on tyrosine residues, and the latter is derived from phenylalanine derivatives synthesized in the thyroid and adrenal medulla, respectively. Such hormones are involved in the regulation of energy metabolism rate [21], heart rate and blood pressure [22], nervous system excitability [23], etc., and are essential for maintaining basic life activities. Thyroxine affects gene expression by binding to nuclear receptors. Catecholamine hormones, on the other hand, often bind to membrane receptors for signal transduction through the G protein-coupled receptor (GPCR) system [24].

In addition to the above classification, there are some special hormones, such as prostaglandins and other lipid derivative hormones, which also play irreplaceable roles in specific physiological processes. In general, hormones weave a vast and intricate regulatory network through their unique chemical properties and mechanisms of action, ensuring that every corner of the human body can receive accurate instructions in time and achieve coordination and unity among various systems.

## EFFECTS OF HORMONES ON EXTRACELLULAR VESICLES

The content of this paragraph is summarized in Table 2.

### Amine hormones

#### Melatonin

MT is one of the hormones secreted by the pineal gland. MT has strong neuroendocrine immunomodulatory activity and free radical scavenging antioxidant capacity, and is eventually metabolized in the liver [25]. MT can be used to prevent and treat liver injury and disease [26]. In their study in mice, Zou et al [27] found that MT treatment significantly increased the expression of *miR-18a-5p* in MSCs and their derived EVs. This result suggested that MT may enhance the biological activity of MSC-derived EVs by upregulating the expression of *miR-18a-5p*. Furthermore, the researchers compared MT-pretreated MT-EVs with untreated MSC-derived EVs. It was found that MT-EVs significantly improved the survival rate of MLE-12 cells (mouse lung epithelial cells). However, these results have not been confirmed in human studies.

Ozansoy et al [28] used the SH-SY5Y cell line and established an Alzheimer’s disease (AD) model by adding different concentrations of amyloid β (Aβ) to induce a toxic response. The experiments were divided into several groups, including cells only, Aβ only, MT only, and the combination of MT and Aβ. Pretreatment with MT (administered before Aβ treatment) was found to significantly reduce the number of EVs released by the cells. This result was obtained by comparing the release of EVs in the ‘cells only’ group with the ‘MT+Aβ’ group, indicating that MT was effective in reducing the release of EVs when administered before Aβ treatment. However, when MT was applied after Aβ treatment, there was no significant change in the number of EVs. This suggests that the timing of MT action is critical for its effect on the release of EVs.

In the study by Hunsaker et al [29], it was found that the addition of MT significantly enhanced the expression of *miR-21* in EVs in all three human oral cancer cell lines: SCC9, SCC25, and CAL27. In contrast, MT significantly reduced the expression of *miR-155*. This finding suggests that MT may affect EV expression by promoting the transcription of *miR-21* and inhibiting the transcription or stability of *miR-155*.

In the study by Liu et al [30] on mice, it was found that MT could affect the secretion process of EVs in adipocytes by activating phosphatidylinositol 3-kinase/protein kinase B (PI3K/PKB) axis and inhibiting the activity of glycogen synthase kinase 3 (GSK-3). Moreover, MT can also increase the secretion of vesicles by promoting the metabolism of intracellular fatty acids and enhancing the interaction between cells. However, these results have not been confirmed in human studies.

In the study by Tang et al [31], MT acted as an antioxidant and was able to directly scavenge free radicals and reduce oxidative stress. Studies have pointed out that there is significant oxidative stress in pre-eclamptic placentas, and the antioxidant properties of MT may help to mitigate this oxidative damage, thereby affecting the production and nature of EVs released from pre-eclamptic placentas. It was also found that EVs released from human pre-eclamptic placentas failed to cause endothelial activation after MT treatment, suggesting that MT may have altered the function of EVs so that they are no longer toxic.

The regulation of EVs by MT in different tissues demonstrates both conserved mechanisms and differential effects. In general, MT regulates EV function through miRNA cargo remodeling: it upregulates *miR-18a-5p* in mesenchymal stem cells (MSCs) and enhances the protective effect of EVs on lung epithelial cells. MT also enriches pro-survival *miR-21* and inhibition of pro-inflammatory *miR-155* in oral cancer cells. In addition, the antioxidant properties of MT constitute another common mechanism: in the AD model, MT inhibits Aβ-induced neuronal EV release by scavenging free radicals, and this effect is dependent on pretreatment timing (posttreatment is not effective). In preeclamptic placentas, MT reverses the endothelial toxicity of EVs by reducing oxidative stress.

In adipocytes, MT promotes EV secretion in a metabolic pathway-dependent manner by activating the PI3K/PKB axis and inhibiting GSK-3, while it inhibits EV release in neurons. In the AD model, pretreatment with MT alone inhibited EV release, suggesting that the timing of administration is the key variable. From the perspective of regulatory strategy, MT focuses on the remodeling of the miRNA profile of EVs in MSCs/oral cancer, depends on signaling pathway activation in adipocytes, and antioxidative stress is the core mechanism in placenta/neurons. These findings reveal that MT has been implicated in the above studies through single or combined mechanisms of miRNA regulation (e.g., miRNA profile remodeling of MSCs and oral cancer cell EVs), oxidative stress buffering (antioxidant effects in AD models and preeclamptic placentas), and signaling pathway adaptation (PI3K/PKB activation in adipocytes). EV functions and their effects differ among tissue types (nerve/fat/placenta) and intervention timing (such as AD pretreatment), providing mechanistic evidence for specific scenarios such as lung protection and neuroprotection mentioned above.

### Peptide hormones

#### Thyroid-stimulating hormone

Thyroid-stimulating hormone (TSH) is a hormone secreted by the adenohypophysis, which produces TSH. On the one hand, it is affected by the promoting effect of thyrotropin-releasing hormone releasing hormone (TRH) secreted by the hypothalamus; on the other hand, its secretion is promoted of thyroid hormone feedback, and these two effects are mutually antagonistic [32]. In the study by Ma et al [33], human liver-derived HepG2 cells were treated with TSH (4 μM) for 24 h. Isolated EVs were validated and quantified by TEM and western blotting using the EV marker antibodies *CD9* and *CD81*. It was found that the number of EVs was significantly increased in TSH-stimulated HepG2 cells. The proteomic changes of isolated EVs in TSH-stimulated HepG2 cells were further quantified by liquid chromatography-mass spectrometry (LC-MS) analysis. Of 1,728 proteins with quantitative information, 140 proteins were upregulated (fold change≥1.5, p<0.05) compared with unstimulated cells. p<0.05). Seven proteins were downregulated (fold change≤–1.5, p<0.05). These results indicated that TSH treatment significantly increased the production of EVs in HepG2 cells and altered the proteomic characteristics of these vesicles. TSH may play an important role in metabolism, apoptosis and inflammation of hepatocytes by increasing the production of EVs and changing their proteomic profiles.

#### Follicle-stimulating hormone

Follicle-stimulating hormone (FSH) is a hormone secreted by basophils of the anterior pituitary, composed of glycoprotein, and its main role is to promote follicle maturation [34]. Mancuso et al [35] found a significant increase in the abundance of several specific proteins in EVs secreted by Sertoli cells after FSH stimulation, including inhibin α (*INHA*), inhibin β (*INHB*), sodium/potassium pump (*AT1A1*), and others. These proteins are associated with cell-cell adhesion and signaling, suggesting that FSH plays an important role in regulating the secretory function of Sertoli cells.

#### Leptin

Leptin is a hormone secreted by adipose tissue [36], and its content in serum is proportional to the size of the animal’s adipose tissue [37,38]. Leptin acts on receptors located in the central nervous system to regulate biological behavior and metabolism [39]. Giordano et al [40] reported a significant increase in the number of MVBs in human breast cancer cells treated with leptin, such as MCF-7 and MDA-MB-231, which in turn resulted in a significant increase in the release of EVs. It has been found that leptin can regulate the biogenesis of EVs by upregulating the protein expression of tumor susceptibility gene 101 (*TSG101*).

### Steroid hormones

#### Estrogen

Estrogen is a substance that promotes the development of secondary sexual characteristics and the maturation of sexual organs in female animals. It is secreted by the ovaries and placenta of female animals. Natural estrogens mainly include estradiol, estrone, and estriol [41]. Drula et al [42] reported that 17β-estradiol regulated the release of EVs in human breast cancer patient-derived breast cancer cells by downregulating the expression of *miR-149-5p*, with a corresponding increase in the number of EVs as estrogen concentration increased. Moreover, after 17β-estradiol treatment, specific miRNAs were enriched in EVs, especially those of the let-7 family, such as *let-7a-5p* and *let-7d-5p*. The levels of these miRNAs were significantly higher in EVs from premenopausal ER+ breast cancer patients than those of postmenopausal patients.

Gonzalez et al [43] found that the addition of 17β-estradiol increased the secretion of EVs from human breast spheroids, and that hormone-treated EVs differed in size and morphology.

#### Testosterone

Testosterone (T) is a steroid hormone, that is secreted by the testes in men or the ovaries in women, and a small amount of T is also secreted by the adrenal glands. It has the functions of maintain muscle strength and mass, maintaining bone density and strength, invigorating and improving physical fitness [44]. In the study by Mancuso et al [35], an increase in the abundance of *INHA*, *INHB*, tissue plasminogen activator (TPA), and epidermal growth factor-like protein 8 (*EGFL8*) was observed in large white pig-derived Sertoli cell extracellular vesicles (SC-EVs) after T stimulation. This indicates that T also has a regulatory role in the secretory function of Sertoli cells and may affect spermatogenesis by promoting the release of bioactive molecules in SC-EVs.

#### Dihydrotestosterone

Dihydrotestosterone (DHT) can be produced directly by the testes or by the conversion of androgens (mainly testosterone) as precursors in surrounding tissues [45]. It can promote the normal development of external genitalia and the prostate, play a positive role in the appearance and maintenance of secondary sexual characteristics, and promote the maturation of sperm in the epididymis [46]. Yan et al [47] reported that, in a study of rat cavernous endothelial cells, the concentration of EVs released by endothelial cells increased significantly with increasing DHT concentration. The lowest concentration of EVs was observed in the androgen-free group (NA group), while the highest concentration of EVs was observed in the physiological androgen concentration group (PA group). Moreover, the expression of CD9, CD63, TSG101 and, eNOS in EVs increased with the increase in DHT concentration. This suggests that DHT not only promotes EV release but may also regulate cell-to-cell signaling by affecting the components of EVs. However, these results have not been confirmed in human studies.

In prostate cancer, Martens-Uzunova et al [48] found that DHT altered the heterogeneity and size of extracellular vesicles (S-EVs) in CD9-positive prostate cancer cells in patients with benign prostatic hyperplasia, as well as in androgen receptor (AR)-expressing prostate cancer cells such as LNCaP cells. DHT treatment altered the size distribution of S-EVs in LNCaP cells, increasing the number of 50-nm diameter s-EVs and also inducing the formation of larger S-EVs. DHT increased the abundance of CD63^+^S-EVs, but decreased the abundance of CD9^+^CD63^+^S-EVs and PSMA+S-EVs. Among the changes in the type and quantity of small RNAs, hormonal stimulation had a strong and specific effect on the small RNA cargo of S-EVs. Androgen increased the levels of a variety of small RNAs (such as *miR-19-3p* and *miR-361-5p*). These include some snRNAs, sdRNAs, tRFs, and piRNAs.

In the study by Salehi et al [49], DHT treatment of rat preantral follicle granulosa cells for 36 h increased the amount of *miR-379-5p* in the EVs of preantral follicle granulosa cells, This suggests that DHT specifically induces the release of *miR-379-5p* from EVs of preantral follicle granulosa cells, but reduces the release of EVs from antral follicle granulosa cells. However, these results have not been confirmed in human studies.

#### Glucocorticoid

Glucocorticoids are an extremely important class of regulatory molecules in the body [50], which play an important role in regulating the development, growth, metabolism, and immune function of the body, and is the most important regulatory hormones of the body’s stress response [51]. Chen et al [52] found that lipopolysaccharide (LPS)-induced secretion of EVs in mouse-derived RAW264.7 macrophages was almost double that of control cells, but this secretion was significantly decreased after dexamethasone (DEX) treatment. The EVs isolated from LPS-induced RAW264.7 cells with or without DEX treatment were then used to treat RAW264.7 cells. These EVs were found to increase the production of TNF-α and IL-6 in RAW264.7 cells, but when treated with EVs isolated from DEX-treated cells, this increase was lower. Additionally, it was also found that glucocorticoid treatment significantly reduced the expression of pro-inflammatory *miR-155* in EVs. Overall, glucocorticoids significantly inhibited LPS-induced TNF-α and IL-6 production by reducing the secretion of EVs and decreasing the expression of *miR-155*.

### Cortisol

Cortisol is a steroid hormone produced by the adrenal glands in response to stress. Cortisol is particularly important in modulating the relationship between mood and health, immune cells and inflammation, blood vessels and blood pressure [53], and in maintaining connective tissue [54]. In times of stress, cortisol generally keeps blood pressure stable and controls inflammation [55]. In the study by Faught et al [56], it was found that in rainbow trout, cortisol treatment significantly reduced HSP70 content in EVs released from hepatocytes. In addition, cortisol treatment also significantly reduced the expression of beta-actin in EVs. This suggests that cortisol not only affects HSP70 expression but may also affect the overall composition and release mechanisms of EVs. However, these results have not been confirmed in human studies.

Steroid hormones—including estrogen, testosterone, DHT, glucocorticoids, and cortisol—exhibit distinct mechanisms in regulating EVs across diverse tissues. Estrogen (17β-estradiol) downregulates *miR-149-5p* in breast cancer cells, increasing EV release and enriching the cargo with let-7 family miRNAs (e.g., *let-7a-5p*), a mechanism linked to premenopausal ER+ tumor progression. Testosterone upregulates *INHA*, *INHB*, and *EGFL8* in Sertoli cell EVs, promoting spermatogenesis via enhanced cell adhesion signaling. DHT, a potent androgen, increases endothelial EV secretion in a concentration-dependent manner (e.g., CD9/CD63 upregulation in rat cavernous cells) while altering prostate cancer EV heterogeneity—boosting 50-nm CD63+ EVs and *miR-19-3p* cargo but reducing CD9^+^/PSMA^+^ subtypes. Glucocorticoids (e.g., dexamethasone) suppress LPS-induced EV secretion in macrophages by 50%, attenuating *miR-155* and TNF-α/IL-6 production, whereas cortisol reduces hepatocyte EV HSP70 and β-actin content in fish models. These findings highlight how steroid hormones leverage EVs to execute tissue-specific functions: estrogen and DHT promote tumorigenesis, testosterone supports spermatogenesis, and glucocorticoids resolve inflammation, and cortisol modulates hepatic stress responses.

## EFFECTS OF EXTRACELLULAR VESICLES ON HORMONES

The content of this paragraph is summarized in Table 3.

### Reproductive system related

#### Mesenchymal stem cells

Human umbilical cord mesenchymal stem cells (HUCMSCs) are multifunctional stem cells that are present in the neonatal umbilical cord tissue. HUCMSCs-EVs have broad application prospects in medical research and treatment because they have a variety of biological functions, including regulating immune responses, promoting tissue repair and regeneration, and inhibiting inflammation and tumor growth [57]. Cai et al [58] found that EVs derived from HUCMSCs are enriched in *miR-21* and can significantly significantly promote estrogen (E_2_) secretion in ovarian granulosa cells (KGN and SVOG cells). This process is mainly achieved by downregulating *LATS1* expression, since overexpression of *LATS1* inhibits estrogen secretion. In addition, EVs also affect the phosphorylation status of *LOXL2* and *YAP* by regulating the expression of *LATS1*, and phosphorylated *LOXL2* and *YAP* inhibit the expression of genes related to estrogen synthesis, such as Steroidogenic acute regulatory protein (*StAR*).

Thus, EVs regulate hormone synthesis and secretion by carrying specific miRNAs. Lu et al found that treatment with HUCMSC-EVs could significantly increase the levels of E_2_ and AMH in rats with primary ovarian insufficiency (POI), while reducing the level of FSH, indicating that EVs can improve ovarian function and promote normal hormone secretion. This result was also confirmed in the experiments of Zhang et al [59]. E_2_ and AMH are important hormones for assessing ovarian function. The increase in E_2_ is associated with follicle maturation and fertility, while the decrease of FSH reflects the recovery of ovarian function. Thus, EVs help restore ovarian function in POI rats by regulating these hormone levels. In addition, HUCMSC-EVs can inhibit oxidative damage and apoptosis of granulosa cells by carrying miR-145-5p, thereby indirectly affecting hormone secretion. The health status of granulosa cells is directly related to the synthesis and secretion of ovarian hormones [60]. However, these results have not been confirmed in human studies.

Meanwhile, in the study by Yang et al [61], it was demonstrated that hormone levels were significantly improved in rats with chemotherapy-induced ovarian failure (POF) after treatment with bone marrow mesenchymal stem cells (BMSCs) or BMSC-derived EVs. Specifically, E_2_ and AMH levels were significantly increased in the treatment group, while FSH and luteinizing hormone (LH) levels were significantly decreased [61]. However, these results have not been confirmed in human studies.

In addition, in the study by Li et al [62], HUCMSC-EVs were able to improve ovarian function in naturally aging (NOA) mice, and this improvement was reflected by restoring hormone levels. Specifically, AMH and E_2_ levels were significantly increased, while FSH levels were significantly decreased in NOA mice after EV treatment. This trend indicated that the levels of AMH and E_2_ in NOA mice were significantly lower than those in young mice, while the level of FSH was significantly higher. After treatment with EVs, the levels of AMH and E_2_ increased, and the level of FSH decreased. However, these results have not been confirmed in human studies.

The results that HUCMSC-EVs can increase the levels of glucocorticoids and AMH and reduce the level of FSH have also been confirmed in another paper by Li et al [63] and in the study by Bianling Xu et al [64]. In addition, in the cisplatin (CDDP)-induced ovarian injury model, HUCMSC-EVs treatment group showed higher E_2_ and AMH levels, indicating that it could effectively reduce the damage caused by chemotherapy drugs to ovarian function. Mechanistic studies have shown that HUCMSC-EVs can improve the survival rate and hormone secretion ability of granulosa cells by regulating the PI3K/Akt signaling pathway and inhibiting the autophagy of granulosa cells, providing a theoretical basis for the role of EVs in the recovery of ovarian function. However, these results have not been confirmed in human studies.

Thabet et al [65] found that in a model of chemotherapy-induced premature ovarian dysfunction (POD), the group treated with amniotic fluid-derived mesenchymal stem cells (AFMSCs) and their derived EVs showed significant recovery of AMH levels. These results suggest that EVs promote the recovery of ovarian function by regulating related biomarkers. In addition, miRNA-21 in EVs inhibits the apoptosis of ovarian cells by downregulating the expression of pro-apoptotic proteins such as *PTEN* and caspase-3, thereby indirectly supporting the normal secretion of AMH. Studies have also found that total ovarian follicle count (TFC) and normal menstrual cycle can be restored after treatment with EVs, which further proves the important role of EVs in maintaining ovarian endocrine function. However, the results have not been confirmed in human studies.

MSCs not only have prominent reparative properties in the ovary, but also play a significant role in the repair of bone marrow and spinal cord. Guo et al [66] found that EVs (synovial mesenchymal stem cell [SMSC]-EVs) secreted by human SMSCs have significant anti-apoptotic effect and can effectively inhibit glucocorticoid-induced apoptosis of BMSCs in a study using rat femoral head necrosis as a model, thereby promoting cell survival and function. In addition, the application of EVs can also reverse the inhibitory effect of glucocorticoids on bone marrow cells and improve Glucocorticoids-induced bone tissue damage, which is specifically manifested as improved the microstructure of bone trabeculae, increased bone mineral density, and reduced the accumulation of adipocytes in bone marrow. However, these results have not been confirmed in human studies.

In the study by Nakazaki et al [67], after Spinal Cord Injury (SCI), the expression level of growth hormone receptor (*GHR*) was significantly decreased in the livers of injured rats, while the level of insulin-like growth factor 1 (*IGF-1*) was also significantly decreased. After injection of human MSC-derived small EVs (HMSC-EVs), the downregulation of *GHR* was significantly improved, and *IGF-1* levels were also restored. This suggests that HMSC-EVs are able to enhance GH signaling by promoting *GHR* expression and indirectly affecting *IGF-1* production. In addition, the levels of proinflammatory cytokines such as TNF-α and IL-6 are significantly increased after SCI, and the increase in these factors is associated with the down-regulation of *GHR* and the reduction of *IGF-1*. HMSC-EVs can help restore the normal hormone signaling pathway by reducing the levels of proinflammatory factors, thereby promoting growth [67]. Thus, EVs play an important role in regulating local and systemic inflammatory responses, affecting the activity of GH and its downstream signaling pathways, and thereby positively affecting growth and metabolism.

#### Follicular fluid

Follicular fluid EVs play an important role in the process of follicular development [68], affecting follicular development and oocyte maturation by regulating gene expression in oocytes [69,70]. In the study by Yuan et al [71], it was found that follicular fluid EVs significantly promoted the synthesis of steroid hormones in porcine theca cells (TCs), especially the levels of progesterone and androstenedione. EVs up-regulated steroid synthesis genes (e.g., *CYP11A1* and *HSD3B1*), which encode key enzymes for progesterone and androstenedione production. Further support for a role for EVs in regulating hormone synthesis is provided. In addition, EVs also promote the proliferation of TCs and indirectly affect the synthesis of steroids by enhancing the proliferative ability of these cells. Studies have found that the proportion of TCs treated with EVs in the S phase of the cell cycle increases significantly, which is closely related to the enhanced hormone synthesis capacity.

The above results were also confirmed in the study by Ying et al [72], in which EVs in bovine small follicular fluid were able to significantly promote steroid hormone synthesis in ovarian cortical stromal cells, especially androstenedione and progesterone. When the concentration of EVs was 30 μg/mL or 100 μg/mL, the level of hormone synthesis was significantly higher than that in the control group (p<0.05). Moreover, EVs regulate the expression of genes involved in steroid hormone synthesis, such as *STAR*, *CYP11A1*, 3β-hydroxysteroid dehydrogenase (3β-HSD), and *CYP17A1*, which play a role. Meanwhile, EVs also promote cell proliferation and inhibit apoptosis, providing a more favorable cellular environment for hormone synthesis.

At the same time, Ying et al [73] found that EVs released from granulosa cells in bovine follicular fluid could significantly promote the secretion of estradiol by granulosa cells, especially at a concentration of 100 μg/mL (p<0.05), but had no significant effect on the secretion of progesterone (p>0.05). In addition, EVs regulate the expression of genes involved in steroidogenesis, promoting the expression of genes such as *CYP19A1* (encoding aromatase, which is responsible for the conversion of androgens into estrogens) and repressing the expression of genes such as *STAR* and HSD3B, potentially affecting progesterone synthesis. It has also been found that EVs may regulate steroid hormone synthesis in granulosa cells through the PI3K/Akt signaling pathway, indicating that EVs play an important regulatory role in the process of hormone synthesis.

#### Semen

In the study by Mateo-Otero et al [74], progesterone (P_4_) and E_2_ secreted by cumulus cells (CCs) during *in vitro* maturation (IVM) were evaluated, and the results showed that supplementation with porcine sperm-derived EVs, did not significantly affect the levels of hormone secretion by CCs. However, EVs significantly altered the expression of genes involved in steroid synthesis, such as *CYP11A1* and *HSD3B1*, suggesting that EVs may indirectly affect steroid hormone synthesis by regulating the expression of these genes. The *CYP11A1* and *HSD3B1* genes encode key enzymes for steroidogenesis, respectively, which are involved in the synthesis of progesterone and other steroid hormones from cholesterol. Despite the effect of EVs on the expression of these genes, the final hormone secretion levels did not change significantly, which may be related to the complex regulatory mechanisms of hormone synthesis.

Mesenchymal stem cell-derived EVs (MSC-EVs) and EVs in follicular fluid, semen, and other body fluids regulate hormone secretion through common mechanisms and tissue-specific strategies. In general, MSC-EVs carrying *miR-21* downregulate *LATS1* to activate the *YAP*/*StAR* axis to promote ovarian estrogen synthesis, and inhibit oxidative stress in granulosa cells to maintain E_2_/AMH secretion through *miR-145-5p*. Both of these processes rely on the PI3K/Akt pathway to inhibit apoptosis or autophagy. Follicular fluid EVs promote the production of progesterone and androstenedione by upregulating steroid synthase genes such as *CYP11A1* and *HSD3B1*. Regarding differences, MSC-EVs significantly increase E_2_/AMH and decrease FSH in the model of premature ovarian failure, and regulated growth hormone signaling by restoring the *GHR*/*IGF-1* axis in SCI. Follicular fluid EVs regulate E_2_ secretion from granulosa cells in a concentration-dependent manner (significant at 100 μg/mL). Although semen EVs regulate *CYP11A1*/*HSD3B1* gene expression, they do not significantly alter hormone levels due to complex feedback mechanism. These findings suggest that EVs can regulate hormonal homeostasis through miRNA profile remodeling (such as *miR-21*/*miR-145-5p* regulation in MSC-EVs), core signal activation (such as anti-apoptotic and proliferative effects mediated by the PI3K/Akt pathway), and tissue microenvironment adaptation (such as ovary/bone marrow Follicular fluid-specific responses) on hormonal homeostasis, providing direct mechanistic evidence for EV-targeted therapy for ovarian function reconstruction (POI/POF models), endocrine disorders, and other diseases mentioned above.

### Metabolic diseases related

#### Hepatoma cell line HepG2

In Mahmoudi-Aznaveh et al [75], it was found that human liver-derived EVs were able to regulate the upregulation of key transcription factors such as Pdx1 and NeuroD1 by stimulating the expression of the insulin gene (Ins1) in pancreatic β cells, thereby promoting insulin synthesis and secretion. In addition, EVs may also further enhance the function of pancreatic β-cells by improving their survival and proliferative ability, especially under conditions of insulin resistance. As carriers of intercellular signaling, these EVs are able to transfer bioactive molecules (such as miRNAs and proteins) released by liver cells to pancreatic beta cells, affecting their hormone synthesis and secretion mechanisms. These results suggest that EVs play an important role in regulating insulin secretion and β-cell function.

#### Breast cancer cell line MCF-7 cells

In the study by Sun et al [76], it was found that human breast cancer cell-derived exosomes significantly promoted lipolysis and inhibited lipogenesis by regulating hormone levels in adipocytes. Specifically, the protein levels of phosphorylated hormone-sensitive lipase (P-HSL) and adipose triglyceride lipase (ATGL) were significantly increased in adipocytes after exosome treatment, indicating that the lipolysis process was promoted, and subsequently, fatty acid release and energy metabolism were increased. At the same time, the expression of the adipogenic markers PPARγ (peroxisome proliferator-activated receptor γ) and AdipoQ (adiponectin) was significantly decreased, suggesting that adipogenesis was inhibited, leading to adipose tissue loss. In addition, the RT-qPCR results showed that the level of the brown adipocyte marker *UCP1* (uncoupling protein 1) was increased, while the level of the white adipocyte marker leptin was decreased in the exosome-treated adipocytes, further supporting the role of exosomes in promoting the conversion of white adipocytes to brown adipocytes. These findings indicate that exosomes regulate the metabolic state of adipocytes and affect hormone secretion and mechanisms of action by transporting *miR-155*, which may be closely related to the development of cancer cachexia, highlighting the important role of exosomes in the regulation of energy metabolism and endocrine function. However, these results have not been confirmed in human studies.

EVs derived from hepatocellular carcinoma cells (HepG2) and breast cancer cells MCF-7 affect hormone homeostasis through common vesicular carrier functions and distinct regulatory pathways: Both of them achieve intercellular information transmission via bioactive molecules such as miRNA and proteins carried by EVs. HepG2-EVs deliver functional molecules to pancreatic β cells and directly upregulate insulin gene Ins1 and transcription factors Pdx1/NeuroD1 to promote insulin synthesis and secretion. They also enhance the survival and proliferative capacity of β cells under insulin-resistant conditions, In contrast MCF-7-EVs delivery *miR-155* to adipocytes promotes lipololysis by activating hormone-sensitive lipase (P-HSL/ATGL) while inhibiting adipogenesis-related genes (PPARγ/AdipoQ), and induce white fat to brown fat conversion (*UCP1*↑/leptin↓). Regarding regulatory pathway, HepG2-EVs directly act on the insulin secretion axis of the endocrine system and reshape hormone synthesis mechanisms through the transcription factor network, while MCF-7-EVs targeted the metabolic-endocrine crossover network and indirectly affected the secretion of hormones such as leptin by reshaping the metabolic state of adipocytes including lipolysis and differentiation. These findings reveal that EVs, as intercellular communication carriers, have two regulatory modes in metabolic diseases: directly regulating genes related to hormone synthesis (such as the regulation of HepG2-EVs on insulin) and indirectly affecting hormone networks through metabolic reprogramming (such as the regulation of MCF-7-EVs on the hormone responsiveness of adipocytes).

### Cancer is linked to hormonal signaling

#### Prostate cancer cells

Prostate cancer cell EVs contain various types of functional cargo, such as nucleic acids, proteins, lipids, and metabolites. These vesicles play a key role in cell-to-cell communication and are able to transfer biologically active molecules into target cells or trigger receptor-mediated signals. In the study by Lei et al [77], it was found that EVs from androgen-independent prostate cancer cells (LNCAP-AI+F) can promote the androgen-independent conversion of androgen-dependent prostate cancer cells (LNCaP), which is associated with the transfer of *let-7a-5p* in EVs. The upregulation of *let-7a-5p* expression promotes the activation of the *AR* signaling pathway. In contrast, EVs derived from androgen-dependent prostate cancer cells were able to reverse the androgen-resistant properties of androgen-independent prostate cancer cells, indicating that EVs from different sources play different roles in regulating the androgen response of prostate cancer cells. In addition, EVs affect the proliferation and transformation of prostate cancer cells by regulating the *AR* and PI3K/Akt signaling pathways. The overexpression of *let-7a-5p* can increase the expression of *AR* and PSA proteins, while its downregulation leads to a reduction in *AR* expression, thereby affecting the androgen dependence of the cells. Overall, EVs have a significant effect on the proliferation of prostate cancer cells, with EVs from androgen-independent cells promoting the proliferation of androgen-dependent cells, while EVs from androgen-dependent cells inhibit the proliferation of androgen-independent cells.

### Inter-organ communication via extracellular vesicles

It was discussed in the previous section that EVs may play an endocrine-like role by delivering regulatory molecules to distant tissues. For example, EVs from liver HepG2 cells are transferred to pancreatic β cells to promote insulin synthesis; EVs from breast cancer MCF-7 cells carry *miR-155* to adipocytes to regulate lipolysis and metabolic hormones; EVs from umbilical cord and BMSCs act on the ovary to regulate E_2_ through blood circulation, affecting the secretion of hormones such as AMH; and MT treatment influences on the hormone balance of maternal vascular endothelial cells in preeclampsia placental EVs.

#### Adipocyte-derived extracellular vesicles act on the pancreas to promote insulin secretion

Kulaj et al [78] showed that AdEVs secreted by adipocytes of obese mice can target pancreatic β cells, and the insulinotropic proteins such as GNAQ and PRKACA carried by them can enhance the sensitivity of β cells to glucose through the GPCR/cAMP/PKA signaling pathway. In addition, the protein cargo such as inflammation-related proteins and lipid metabolism enzymes of AdEVs changes in obesity, and their luminal morphology was more easily taken up by β cells. In vitro studies showed that AdEVs significantly increased first-phase insulin secretion (GSIS) in mouse islets. *In vivo* studies confirmed that AdEV pretreatment in obese mice improved glucose tolerance and enhanced insulin secretion [78].

#### Hepatic extracellular vesicles regulate blood glucose homeostasis in metabolic organs

In the study by Hu et al [79], and Miotto et al [80], it was noted that EVs secreted by the liver can affect pancreatic β-cell function through different mechanisms. On the one hand, hepatic EVs carry specific miRNAs or proteins such as miR-122, which inhibit the glycolysis-related gene PKM after acting on pancreatic β-cells, thereby inhibiting insulin secretion. In the obese state, the cargo change of hepatic EVs may conversely promote insulin secretion, thereby contributing to the regulation of blood glucose homeostasis. On the other hand, hyperglycemia stimulates the liver to increase the secretion of EVs, which directly promotes glucose oxidation and glucose-stimulated insulin secretion (GSIS) of β-cells independent of insulin sensitivity, after reaching the pancreas through the blood. Moreover, this process enhances the β-cell response to glucose by activating the CaMKII signaling pathway, resulting in increased insulin secretion.

## NON-CODING RNAs IN EXTRACELLULAR VESICLES: REGULATORS OF KEY ENZYMES FOR HORMONE SYNTHESIS

The mechanism by which ncRNA directly targets enzyme genes to regulate key enzymes of hormone synthesis has been detailed in the previous article. Among them, *miR-21* (HUCMSC-EVs) activates *StAR* by downregulating *LATS1*, thereby promoting estrogen synthesis [54]. In addition, *miR-379-5p* (DHT-treated follicular granulosa cell EVs) targeted *CYP19A1* (aromatase) and inhibited the conversion process of androgen to estrogen. These mechanisms lay a theoretical foundation for subsequent exploration of the regulatory effects of new ncRNAs. This paragraph is summarized in (Table 4).

### *CDC42* signaling activation mechanism of lncRNA *LOC105611671*

In the study by Wang et al [81], it was found that lncRNA *LOC105611671* in ovarian granulosa cell EVs directly binds to *CDC42* protein to activate PI3K-Akt signaling pathway. The expression of *StAR* and *CYP11A1* (cholesterol side-chain cleavage enzyme) was significantly upregulated. Among them, *StAR* promotes cholesterol transport to mitochondria, and *CYP11A1* catalyzes the conversion of cholesterol to pregnenolone, which is the initial step in steroid synthesis. Functional experiments showed that overexpression of the lncRNA increased estrogen (E_2_) and progesterone (P_4_) secretion by 23% and 18%, respectively.

### Dual inhibition of granulosa cell enzyme activity by miRNA in polycystic ovary syndrome

In the study by Zhang et al [82], it was found that *miR-143-3p* in polycystic ovary syndrome (PCOS) follicular fluid EVs targeted *BMPR1A* and inhibited Smad1/5/8 signaling, resulting in a 40% increase in the granulosa cell apoptosis rate and indirectly reducing *CYP19A1* expression. It also reduced E_2_ synthesis by 31%. In addition, *miR-320a* in serum EVs of PCOS increased free testosterone concentration by 55% by inhibiting the p85/PI3K-Akt pathway, exacerbating insulin resistance, activating *CYP17A1* (17α-hydroxylase) in the ovary, and inhibiting SHBG synthetase in the liver.

### *lncGSAR* regulates steroid synthetase via the ceRNA network

In the study by Wang et al [83], it was found that *lncGSAR* in ovarian granulosa cell EVs acts as a competitive endogenous RNA (ceRNA), sponging miR-125b to relieve its inhibition of SCAP (SREBP cleavage-activating protein) and activate the SCAP/SREBP pathway. This pathway significantly upregulated the transcription of *CYP11A1* (↑41%) and *CYP19A1* (↑35%), and promoted the expression of *StAR* (↑28%), resulting in a 52% increase in E_2_ secretion and a 27% decrease in P_4_ secretion.

### Future research directions

Current studies have revealed the bidirectional regulatory network between hormones and EVs, but there are still three key gaps that need to be further explored:

First, the expansion of the regulatory mechanisms of neuroendocrine and metabolic hormones. Although MT can affect cell function by regulating miRNAs (such as miR-18a-5p) in EVs, the systemic regulatory mechanisms of EVs on thyroid hormones (T3/T4), leptin and other neuroendocrine and metabolic hormones remain unclear. It is necessary to explore whether functional molecules (such as miRNAs and proteins) carried by EVs participate in the regulation of hormone synthesis and secretion by targeting endocrine organ receptors (such as the thyroid receptor and leptin receptor), thereby providing new targets for metabolic syndrome and neurodegenerative diseases.

Second, the precise elucidation of the molecular mechanism of bidirectional regulation. Hormones (such as estrogen and testosterone) regulate the specific nodes of EV biogenesis through the PI3K/Akt, Hippo-YAP, and other pathways, and molecules such as let-7 family miRNAs and YAP carried by EVs counterregulate the key steps of hormone signaling. This still needs to be further verified by single-cell sequencing and gene editing technologies. In addition, the specific molecular events of hormone-EV interaction under pathological conditions (such as tumors and preeclampsia) also need to be further elucidated in organoids and animal models.

Third, the key breakthrough in clinical translation and application. Developing non-invasive diagnostic tools based on hormone-regulated EV markers, such as let-7a-5p in breast cancer EVs and miR-19-3p in prostate cancer EVs. At the same time, the drug delivery system of hormone-modified EVs (such as glucocorticoid-modified EVs for anti-inflammatory targeted therapy) is being optimized, and the clinical potential of HUCMSC-EVs in ovarian function repair is being explored, in order to translate the basic mechanisms into a precise diagnosis and treatment strategy.

The abbreviations and full name of the nouns in the text is summarized in Table 5.

## CONCLUSION

Taken together, this review discusses the interactions between EVs and hormones. This paper focuses on the effects of hormones on EVs, such as MT regulating miRNAs in EVs, estrogen altering EV release, and glucocorticoids inhibiting inflammation-related EV secretion. At the same time, we analyzed the regulation of hormones by EVs, such as MSC-derived EVs promoting ovarian estrogen secretion and follicular fluid EVs promoting steroid hormone synthesis, as well as the role of non-coding RNAs in EVs in regulating key enzymes involved in hormone synthesis. In the future, it is necessary to further explore the molecular mechanisms and directions for clinical translation, which will provide a theoretical basis and clinical insights for the study of animal physiological regulation.

## Figures and Tables

**Table 1:** Origin and classification of EVs

Type	Mechanism of formation	Characteristics	Function
Exosomes	Derived from MVBs formed by endocytosis. MVBs fuse with cell membrane, releasing internal vesicles. Process regulated by endosomal sorting mechanisms.	Diameter about 40–150 nm. Rich in unique lipids, proteins (e.g., tetraspanins, HSP70) and nucleic acid molecules (e.g., various miRNAs).	Participate in intercellular signal transmission. Affect physiological state of adjacent or distal cells. Promote immune response, nerve signal transduction, tumor microenvironment remodeling, etc.
Microvesicles	Formed by budding of cell membrane. Related to cell activation, injury, or stress. Formation related to asymmetric distribution of phospholipids in cell membrane. Calcium influx and other factors promote budding.	Diameter mostly 100–1,000 nm. Membrane composition similar to that of the parent cell.	Associated with cell states. Considered as a mechanism for the repair of plasma membrane damage.
Apoptotic bodies	During apoptosis, cytoplasm is fragmented and packaged into large vesicles, then released from the cell. Cell membrane shrinks and invaginates, wrapping cell the contents.	Diameter 500–2,000 nm (50–5,000 nm in some literatures).	Play a key role in clearing senescent or damaged cells, avoiding inflammatory responses, maintaining tissue stability.

EVs, extracellular vesicles; MVBs, multivesicular bodies; HSP70, heat shock protein 70.

**Table 2:** Effects of hormones on EVs

Hormone	Effects on EVs	Reference
Melatonin	MT raises *miR-18a-5p* levels in MSCs and their exosomes, and MT-EVs boost MLE-12 cell survival. Melatonin pretreatment reduces cell-released EVs. It regulates miR-21 and *miR-155* in oral cancer exosomes, impacts adipocyte extracellular vesicle secretion, and alters those from pre-eclamptic placentas.	[27–31]
Estrogen	17β-estradiol adjusts EV release in breast cells, enriches EVs, and alters EV traits.	[42,43]
Follicle stimulating hormone	FSH increases protein levels in SC-EV, and impacts cell-related functions.	[35]
Testosterone	T increases protein abundance in pig SC-EV, and regulates SC secretion.	[35]
Dihydrotestosterone	In rat endothelial cells, promotes EV release & protein levels. In prostate cancer, modifies S-EVs traits & small RNAs. In follicle cells, adjusts *miR-379-5p* and extracellular vesicle release.	[47–49]
Leptin	In breast cancer cells, raises multivesicular bodies, increasing EV release & regulating biogenesis via *TSG101*.	[40]
Thyroid-stimulating hormone	In HepG2 cells, increases EV production & modifies proteomic traits.	[33]
Glucocorticoids	In macrophages, reduces EV secretion, decreases TNF-α, IL-6, and *miR-155* levels.	[52]
Cortisol	In rainbow trout hepatocytes, reduces HSP70 and beta-actin in EVs.	[56]

EVs, extracellular vesicles; MT, melatonin; MSC, mesenchymal stem cell; FSH, Follicle-stimulating hormone; SC-EV, Sertoli cell extracellular vesicle; T, testosterone; HSP70, heat shock protein 70.

**Table 3:** Effects of EVs on hormones

Types of EVs	Source	Effects on hormones	Reference
HUCMSC-EVs	Human umbilical cord mesenchymal stem cells	Promote E_2_ secretion in ovarian granulosa cells; increase E_2_, AMH, Granulosa Cells levels, and decrease FSH, LH levels in multiple ovarian models	[58–65]
SMSC-EVs	Human synovial mesenchymal stem cells	Alleviate bone marrow-related hormone-induced damage by inhibiting BMSC apoptosis	[66]
HMSC-EVs	Human bone marrow mesenchymal stem cells	Increase *GHR* expression and restore IGF-1 level in SCI rats	[67]
EVs derived from AFMSC	Amniotic fluid-derived mesenchymal stem cells	Restore AMH level in POD model	[65]
EVs derived from BMSC	Bone marrow mesenchymal stem cells	Increase E_2_, AMH levels and decrease FSH, LH levels in POF rats	[61]
Follicular fluid EVs	Follicular fluid	Promote steroid hormone synthesis in porcine and bovine ovarian cells; boost E_2_ secretion in bovine granulosa cells	[71,72]
Porcine sperm-derived EVs	Porcine sperm	Alter steroid-related gene expression in CCs, no significant effect on P_4_ and E_2_ secretion	[74]
Prostate cancer cell EVs	Prostate cancer cells	Affect prostate cancer cell androgen-related hormone mechanisms via AR and PI3K/Akt pathways	[77]
Human liver-derived EVs	Human liver (HepG2 cells)	Promote insulin synthesis and secretion in pancreatic β-cells	[75]
Exosomes derived from human breast cells	Human breast cancer cells (MCF-7 cells)	Regulate adipocyte hormone levels, promote lipolysis and inhibit lipogenesis	[76]

EVs, extracellular vesicles; HUCMSC, human umbilical cord mesenchymal stem cell; AMH, anti-Müllerian hormone; FSH, Follicle-stimulating hormone; LH, luteinizing hormone; SMSC, synovial mesenchymal stem cell; BMSC, bone marrow mesenchymal stem cell; HMSC, human mesenchymal stem cell; IGF-1, insulin-like growth factor 1; SCI, spinal cord injury; AFMSC, amniotic fluid-derived mesenchymal stem cell; POD, premature ovarian dysfunction; POF, premature ovarian failure; CC, cumulus cell; AR, androgen receptor.

**Table 4:** Regulatory effects of ncRNAs in EVs on key enzymes of hormone synthesis

ncRNA Type	Specific molecule	EV source	Regulated enzyme/signaling pathway	Hormone synthesis effect	Reference
miRNA	*miR-21*	HUCMSC-EVs	*LATS1*→*StAR*	Increased E_2_ synthesis	[58]
miRNA	*miR-379-5p*	DHT-treated follicular granulosa cell EVs	*CYP19A1*	Reduced androgen-to-estrogen conversion	[49]
miRNA	*miR-143-3p*	PCOS follicular fluid EVs	*BMPR1A*→*CYP19A1*	31% decrease in E_2_ synthesis	[82]
miRNA	*miR-320a*	PCOS serum EVs	p85/PI3K-Akt→*CYP17A1*/SHBG synthase	55% increase in free testosterone	[82]
lncRNA	*LOC105611671*	Ovarian granulosa cell EVs	*CDC42*→PI3K-Akt→*StAR/CYP11A1*	23% increase in E_2_ synthesis, 18% increase in P_4_ synthesis	[81]
lncRNA	*lncGSAR*	Ovarian granulosa cell EVs	miR-125b→SCAP→*CYP11A1/CYP19A1*	52% increase in E_2_ synthesis, 27% decrease in P_4_ synthesis	[83]

EVs, extracellular vesicles; HUCMSC, human umbilical cord mesenchymal stem cell; DHT, dihydrotestosterone; PCOS, polycystic ovary syndrome.

**Table 5:** Summary of abbreviations and full name

Abbreviations	Full name	Abbreviations	Full name
EVs	Extracellular vesicles	YAP	Yes-associated protein
MVBs	Multivesicular bodies	StAR	Steroidogenic acute regulatory protein
HSP70	Heat shock protein 70	POI	Primary ovarian insufficiency
MT	Melatonin	AMH	Anti-müllerian hormone
MSCs	Mesenchymal stem cells	POF	Premature ovarian failure
MLE-12 cells	Mouse lung epithelial cells	BMSCs	Bone marrow mesenchymal stem cells
AD	Alzheimer’s disease	LH	Luteinizing hormone
Aβ	Amyloid β	NOA	Naturally aging
PI3K/PKB	Phosphatidylinositol 3-kinase/protein kinase B	CDDP	Cisplatin
GSK-3	Glycogen synthase kinase 3	AFMSCs	Amniotic fluid-derived mesenchymal stem cells
TSH	Thyroid-stimulating hormone	POD	Premature ovarian dysfunction
TRH	Thyrotropin-releasing hormone	PTEN	Phosphatase and tensin homolog
LC-MS	Liquid chromatography-mass spectrometry	TFC	Total ovarian follicle count
FSH	Follicle-stimulating hormone	SMSC-EVs	Synovial mesenchymal stem cell-derived EVs
INHA	Inhibin α	SCI	Spinal cord injury
INHB	Inhibin β	GHR	Growth hormone receptor
T	Testosterone	IGF-1	Insulin-like growth factor 1
DHT	Dihydrotestosterone	TCs	Theca cells
TPA	Tissue plasminogen activator	CYP11A1	Cytochrome P450 family 11 subfamily A member 1
EGFL8	Epidermal growth factor-like protein 8	HSD3B1	3β-Hydroxysteroid dehydrogenase 1
eNOS	Endothelial nitric oxide synthase	3β-HSD	3β-Hydroxysteroid dehydrogenase
AR	Androgen receptor	CYP17A1	Cytochrome P450 family 17 subfamily A member 1
S-EVs	Small extracellular vesicles	CYP19A1	Cytochrome P450 family 19 subfamily A member 1
PSMA	Prostate-specific membrane antigen	P^4^	Progesterone
snRNAs	Small nuclear RNAs	Ins1	Insulin 1
sdRNAs	Small DNA-derived RNAs	Pdx1	Pancreatic and duodenal homeobox 1
tRFs	tRNA-derived fragments	NeuroD1	Neurogenic differentiation 1
piRNAs	Piwi-Interacting RNAs	P-HSL	Phosphorylated hormone-sensitive lipase
LPS	Lipopolysaccharide	ATGL	Adipose triglyceride lipase
DEX	Dexamethasone	PPARγ	Peroxisome proliferator-activated receptor γ
TNF-α	Tumor necrosis factor-α	AdipoQ	Adiponectin
IL-6	Interleukin-6	UCP1	Uncoupling protein 1
Hsp70	Heat shock protein 70	AdEVs	Adipocyte-derived EVs
HUCMSCs	Human umbilical cord mesenchymal stem cells	GNAQ	G protein subunit alpha Q
E2	Estradiol	PRKACA	Protein kinase CAMP-activated catalytic subunit alpha
LATS1	Large tumor suppressor 1	GPCR	G protein-coupled receptor
LOXL2	Lysyl oxidase-like protein 2	cAMP	Cyclic adenosine monophosphate

## Data Availability

Upon reasonable request, the datasets of this study can be available from the corresponding author.
